# A Putative Amidase Endolysin Encoded by *Clostridium perfringens* St13 Exhibits Specific Lytic Activity and Synergizes with the Muramidase Endolysin Psm

**DOI:** 10.3390/antibiotics10030245

**Published:** 2021-03-01

**Authors:** Hiroshi Sekiya, Maho Okada, Eiji Tamai, Toshi Shimamoto, Tadashi Shimamoto, Hirofumi Nariya

**Affiliations:** 1 Department of Infectious Disease, College of Pharmaceutical Sciences, Matsuyama University, 4-2 Bunkyo-cho, Matsuyama, Ehime 790-8578, Japan; hsekiya@g.matsuyama-u.ac.jp (H.S.); etamai@g.matsuyama-u.ac.jp (E.T.); 2 Laboratory of Food Microbiology and Hygiene, Graduate School of Biosphere Science, Hiroshima University, 1-4-4 Kagamiyama-cho, Higashihiroshima, Hiroshima 739-8528, Japan; ma-horn@outlook.com; 3 Laboratory of Food Microbiology and Hygiene, Graduate School of Integrated Sciences for Life, Hiroshima University, 1-4-4 Kagamiyama, Higashihiroshima, Hiroshima 739-8528, Japan; tsima@hiroshima-u.ac.jp (T.S.); tadashis@hiroshima-u.ac.jp (T.S.); 4 Laboratory of Food Microbiology, Graduate School of Human Life Sciences Food and Nutritional Sciences, Jumonji University, 2-1-28, Kansawa, Niiza, Saitama 352-8510, Japan

**Keywords:** endolysin, amidase, *Clostridium perfringens*, bacterial cell wall, peptidoglycan

## Abstract

*Clostridium perfringens* is an often-harmful intestinal bacterium that causes various diseases ranging from food poisoning to life-threatening fulminant disease. Potential treatments include phage-derived endolysins, a promising family of alternative antimicrobial agents. We surveyed the genome of the *C. perfringens* st13 strain and identified an endolysin gene, *psa*, in the phage remnant region. Psa has an N-terminal catalytic domain that is homologous to the amidase_2 domain, and a C-terminal domain of unknown function. *psa* and gene derivatives encoding various Psa subdomains were cloned and expressed in *Escherichia coli* as N-terminal histidine-tagged proteins. Purified His-tagged full-length Psa protein (Psa-his) showed *C. perfringens*-specific lytic activity in turbidity reduction assays. In addition, we demonstrated that the uncharacterized C-terminal domain has cell wall-binding activity. Furthermore, cell wall-binding measurements showed that Psa binding was highly specific to *C. perfringens*. These results indicated that Psa is an amidase endolysin that specifically lyses *C. perfringens*; the enzyme’s specificity is highly dependent on the binding of the C-terminal domain. Moreover, Psa was shown to have a synergistic effect with another *C. perfringens*-specific endolysin, Psm, which is a muramidase that cleaves peptidoglycan at a site distinct from that targeted by Psa. The combination of Psa and Psm may be effective in the treatment and prevention of *C. perfringens* infections.

## 1. Introduction

The number of drug-resistant bacteria is increasing worldwide, and the development of new antibacterial drugs is on the decline, a combination that represents a major challenge for the international medical community [1,2,3]. Lytic enzymes kill bacteria by degrading the peptidoglycan mesh of the cell wall. In Gram-positive bacteria, peptidoglycan is exposed on the surface, so externally applied lytic enzymes can readily access the peptidoglycan, resulting in osmotic lysis. Lytic enzymes, especially phage-derived endolysins, are thus a promising family of alternative antimicrobial agents [3,4,5,6]. Several endolysins have been successfully applied in animal models of infection [4,7,8,9,10,11,12,13].

**Figure 1:**
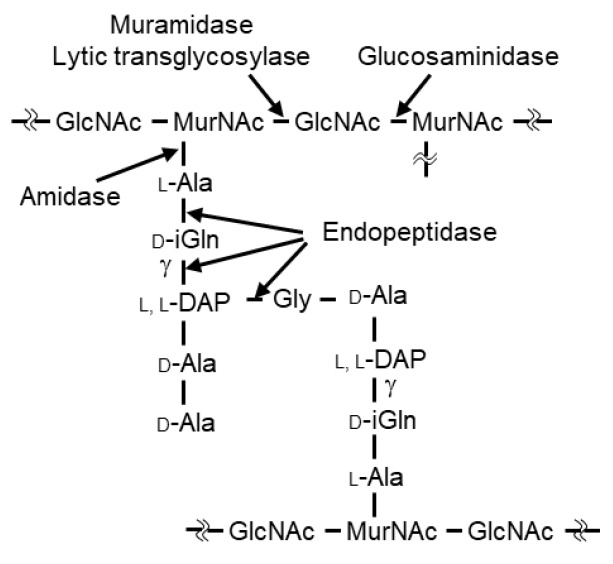
Schematic diagram of the main types of peptidoglycan in C. perfringens (Leyh-Bouille, M., et al.). GlcNAc, N-acetylglucosamine; MurNAc, N-acetylmuramic acid; iGln, isoglutamine; L,L-DAP, L,L-diaminopimelic acid. Hydrolyzing sites are indicated for the five classes of lytic enzymes.

In general, lytic enzymes are composed of a catalytic domain (CD) that hydrolyzes specific sites within peptidoglycan and a cell wall-binding domain (CBD) that specifically recognizes the bacterial cell wall [14]. There are five classes of lytic enzymes (glucosaminidase, muramidase, amidase, endopeptidase, and lytic transglycosylase), reflecting the site targeted for hydrolysis and the structural homology of the proteins [15] (Figure 1). Two well-known lytic enzymes are endolysin and autolysin. Endolysin is a phage-derived lytic enzyme that is expressed in the final stages of phage infection and is transported to the outside of the infected cell by phage-encoded holins. Generally, genes encoding endolysins are present in dual lysis gene cassettes in association with holin genes [16]. The released endolysin promotes the release of progeny phages by hydrolyzing cell wall peptidoglycan [17,18]. Typically, endolysins lack a signal peptide, and the CD is N-terminal while the CBD is C-terminal. The lytic activity of endolysins generally is species specific. The other well-known lytic enzyme, autolysin, is a bacterial lytic enzyme that is involved in various physiological functions such as cell wall remodeling, cell wall expansion, and/or daughter cell separation [19,20,21]. Autolysins generally have low species specificity.

*Clostridium perfringens* is a spore-forming Gram-positive anaerobe that is widely distributed in nature, especially in soil and the intestinal tracts of humans and animals. *C. perfringens* produces various potent toxins [22,23]. *C. perfringens* strains st13 and SM101, that have been completed the genome sequences and are commonly used in studies of *C. perfringens* pathogenicity, produce α-toxin (phospholipase C, PLC), θ-toxin (perfringolysin O, PFO), κ-toxin (collagenase, ColA), μ-toxin (hyaluronidase), and λ-toxin (clostripain-like protease, CLP). This bacterium is responsible for food poisoning, gas gangrene, and necrotic enteritis, both in humans and animals [24,25,26,27]. Food poisoning caused by *C. perfringens* is among the most common gastrointestinal diseases in industrialized countries [27,28]. The incidence of necrotic enteritis due to the presence of *C. perfringens* in poultry has increased in countries where the use of antibacterial growth promoters in feed has been abolished [29,30]. The use of *C. perfringens*-specific lytic enzymes provides additional opportunities to reduce the infectious diseases caused by *C. perfringens* without disturbing the balance of the microbiota in the gastrointestinal tract.

To facilitate the use of endolysin as a biocontrol and therapeutic agent for infectious diseases caused by *C. perfringens*, the genomes and bacteriophage sequences of *C. perfringens* strains have been surveyed to identify putative endolysin-encoding loci. One such gene is *psm*, which is located on phiSM101, the episomal phage, one of prophages, of enterotoxigenic *C. perfringens* SM101. The Psm protein is a muramidase endolysin that exhibits potent and highly specific lytic activity toward *C. perfringens* [31]. Psm has an N-terminal CD belonging to the glycoside hydrolase 25 [GH25, Pfam number (http://pfam.sanger.ac.uk/) (2 February 2018) PF01183] family of muramidases (enzymes known to hydrolyze between *N*-acetylmuramic acid and *N*-acetylglucosamine) and a C-terminal CBD containing two tandemly repeated bacterial Src homology 3_3 family (SH3_3, PF08239) subdomains [32]. In 2003, two phage-encoded lysins of *Streptococcus pneumoniae* were reported to degrade different sites in the cell wall of the host bacterium and to exhibit a synergistic killing effect on that species [33]. We hypothesized that if Psm and a *C. perfringens*-specific lytic enzyme that hydrolyzes peptidoglycans at a different site from Psm could be used together, a synergistic effect can be expected in *C. perfringens*. Amidase endolysins, which are found in many bacteria and phages (Figure S1), may have synergistic effects with Psm. In particular, the T7 phage amidase endolysin (T7 lysozyme) and the *Escherichia coli* amidase autolysin AmiD have been well studied; their biochemical properties have been clarified [34,35], and the amino acid residues that constitute their active sites and substrate recognition sites have been identified structurally [36,37]. Based on those results, we suspected that investigation of the genomes of various *C. perfringens* strains might be useful for identifying amidase endolysins specific to *C. perfringens*.

Here, we report the discovery, in the genome of *C. perfringens* strain st13, of a gene encoding Psa, a novel endolysin that has an N-terminal amidase_2 domain. We characterized Psa’s biochemical properties, including its species specificity and synergistic effects with the *C. perfringens*-specific muramidase endolysin Psm.

## 2. Results

### 2.1. Identification and Cloning of Psa from the Genome of Clostridium perfringens st13, and Expression and Purification of the Psa Protein

A BLAST search (http://www.ncbi.nlm.nih.gov/BLAST/) (2 February 2018) revealed that a 36.1-kbp putative phage remnant region exists only on the genome of *C. perfringens* st13 and is absent from the genomes of the other sequenced *C. perfringens* strains (Figure S2). A gene designated *psa* (gene ID; CPE1138), encoding a putative endolysin, was detected at one end of this region; a putative holin gene is also present in the vicinity. A domain search (https://pfam.xfam.org/) (2 February 2018) using the predicted amino acid sequence of Psa suggested that this protein contains an N-terminal amidase_2 domain (PF01510). Specifically, the N-terminal domain exhibits 69.8% similarity to the corresponding domain of T7 lysozyme [34]. Analysis with SignalP (http://www.cbs.dtu.dk/services/SignalP/) (2 February 2018) indicated that Psa lacks a signal peptide, like most phage endolysins characterized to date. Therefore, Psa is thought to be an endolysin with amidase activity that cleaves the amide bond between *N*-acetylmuramic acid and L-Ala (Figure 1). Furthermore, amino acid sequence alignment analysis suggested that Tyr51 is involved in the catalytic reaction [36] and that His26, His123, and Cys131 are involved in zinc binding, since the amino acids involved in zinc binding in zinc-dependent amidases such as T7 lysozyme and AmiD are conserved in Psa (Figure S1). The C-terminal region appears to be a novel CBD, given that bioinformatic analysis using BLAST did not reveal any homology with known domain sequences.

**Figure 2:**
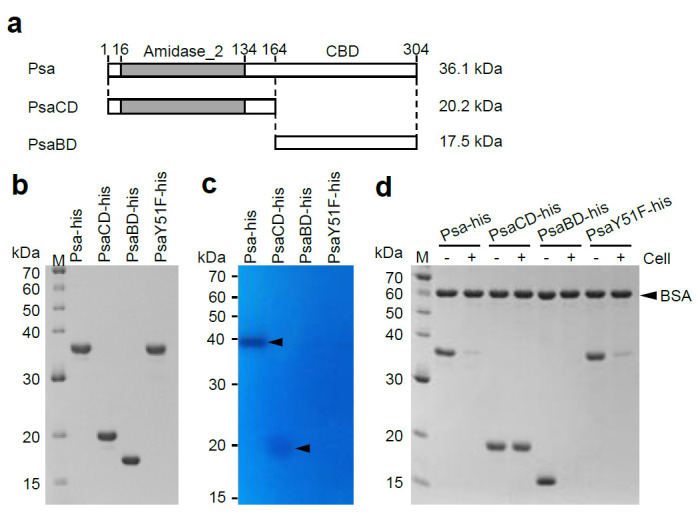
SDS-PAGE analysis, zymography analysis, and binding analysis of Psa-his, PsaCD-his, PsaBD-his, and PsaY51F-his. (**a**) Schematic diagrams of Psa, PsaCD, and PsaBD are shown Psa has an N-terminal catalytic domain (CD) of the amidase_2 family (indicated by gray bar) and a novel C-terminal cell wall-binding domain (CBD). These proteins were expressed and purified with a histidine-tag (MNHKVHHHHHH) fused to the N-terminus. The molecular weights of each histidine-tagged protein are shown on the right of the corresponding horizontal bar. (**b**) Purified Psa-his, PsaCD-his, PsaBD-his, and PsaY51F-his (1 μg each) were subjected to 12.5% SDS-polyacrylamide gel electrophoresis. The gel was stained with Coomassie blue R. (**c**) Zymography analysis was carried out according to the Materials and Methods. Purified Psa-his (0.5 μg), PsaCD-his (2.0 μg), PsaBD-his(2.0 μg), and PsaY51F-his (0.5 μg) were electrophoresed in a 12.5% SDS-polyacrylamide gel containing 20 OD_600_ of heat-inactivated and SDS-treated *C. perfringens* HN1314 cells. The arrowheads indicate cell lysis. (**d**) Binding activity was measured according to the Materials and Methods. The purified protein and non-binding internal standard, bovine serum albumin (BSA), were combined with (+) or without (−) *C. perfringens* HN1314 cells; the mixtures then were centrifuged, and the resulting supernatants were analyzed by 12.5% SDS-PAGE.

Various segments of the *psa* open reading frame (ORF) were cloned into the *E. coli* expression vector pColdII to encode Psa derivatives harboring a N-terminal histidine tag (MNHKVHHHHHH). The predicted fusion proteins included the entire Psa protein (Psa-his), Psa with a mutated catalytic center (PsaY51F-his), the Psa N-terminal region alone (amino acids 1-164; PsaCD-his), and the Psa C-terminal region alone (amino acids 165-304; PsaBD-his) (Figure 2a). The recombinant proteins of Psa-his (315 amino acids, 36,091 Da), PsaY51F-his, PsaCD-his (175 amino acids, 20,219 Da), and PsaBD-his (152 amino acids, 17,453 Da) were successfully expressed and purified by immobilized metal chelate affinity chromatography, and the purities of the final samples were confirmed by SDS-PAGE (Figure 2b).

### 2.2. Characterization of the Enzyme Activity of Psa

To examine the lytic activity of the purified proteins, turbidity reduction assays were performed using several *C. perfringens* strains as substrates. *C. perfringens* HN1314 is a *C. perfringens* st13 derivative in which the following six genes have been disrupted, making the strain safe for laboratory use [38]: Genes encoding the virulence factors PLC, CLP, ColA, and PFO; the gene encoding the major secretory protein CPE1281; and the gene encoding CwlO, a putative cell wall lytic endopeptidase. We investigated whether *C. perfringens* HN1314 could be used in this study by examining the susceptibility of this strain to Psa-his. Psa-his showed marked lytic activity against *C. perfringens* st13 derivative strain HN1314 (3967 U) but showed lower lytic activity against *C. perfringens* st13 (32% of that against HN1314) and SM101 (25% of that against HN1314) (Table 1). Psa-his also showed a clear lytic band in zymographic analysis using *C. perfringens* HN1314 as a substrate (Figure 2c). These data indicated that Psa has lytic activity against *C. perfringens*, and that *C. perfringens* HN1314 is a suitable strain for biochemical analysis of Psa activity. Notably, PsaY51F-his, PsaCD-his, and PsaBD-his did not show any lytic activity against *C. perfringens* HN1314 in the turbidity reduction assay (Figure S3), but a faint lytic band was visible in zymographic analysis when PsaCD-his was added to an approximately 4-fold higher concentration (in µg/mL; corresponding to an approximately 8-fold higher molar concentration) compared to the full-length enzyme (Figure 2c). When the same concentration of Psa-CD as Psa-his was used to the analysis, the lytic band of Psa-CD was quite faint compared with the lytic band of Psa-his (Figure S4). These results suggested that PsaCD-his has weak lytic activity as assessed by zymographic analysis. In addition, to examine the cell binding ability of the purified protein, a cell binding assay was performed using *C. perfringens* HN1314 as the substrate. The cell binding activity was measured by the addition of purified protein to a cell suspension; the suspension was then centrifuged, and the amount of His-tagged protein remaining in the supernatant was assessed by SDS-PAGE. In this assay, the protein band density is reduced compared to a cell-free control if the purified protein has the ability to bind cells. As shown in Figure 2d, the bands of Psa-his, PsaY51F-his, and PsaBD-his disappeared in this cell binding assay, revealing that these proteins are able to bind to *C. perfringens*. These results demonstrated that the N-terminal domain is the CD and that Tyr51 is involved in the catalytic reaction, while the C-terminal domain is the CBD, without which the CD does not have lytic activity in aqueous solution.

**Figure 3:**
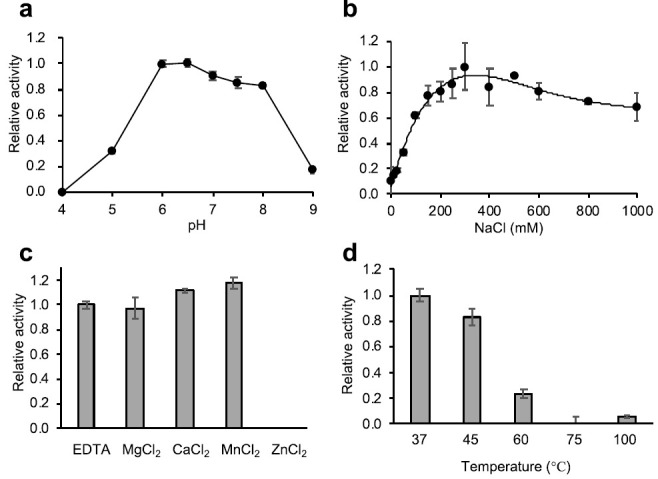
Characterization of the lytic activity of Psa-his. The lytic activity of Psa (1 U) against C. perfringens HN1314 was determined using the turbidity reduction assay. (**a**) Optimal pH of Psa-his. The relative activity at pH 6.0 was set as 1. (**b**) The effect of NaCl on Psa-his lytic activity was determined, and the relative activity at 300 mM NaCl was set as 1. (**c**) The effects of divalent metal cations on Psa-his lytic activity were determined by the addition of 1 mM MgCl_2_, CaCl_2_, MnCl_2_, ZnCl_2_, or EDTA. The relative activities are shown with activity in the absence of divalent cations (with 1 mM EDTA) set as 1. (**d**) The thermal-stability of Psa-his lytic activity was determined by measuring lytic activity after 10 min of heat treatment at 37, 45, 60, 75, and 100 °C. The relative activity at 37 °C was set as 1.

To characterize the enzyme activity of Psa, we determined the effect of pH, salt, metal ions, and temperature on the lytic activity using *C. perfringens* HN1314 as a substrate (Figure 3a–d). As shown in Figure 3a, the optimal pH was between 6 and 8, and the activity significantly decreased at pH values above 9 and below 5. The activity increased with the addition of NaCl, peaking at 300 mM NaCl, and no significant decrease in activity was observed at up to 1000 mM NaCl (Figure 3b). The addition of MgCl_2_, CaCl_2_, MnCl_2_, and ethylenediaminetetraacetic acid (EDTA) did not significantly affect the lytic activity, but the addition of ZnCl_2_ significantly reduced the lytic activity (Figure 3c). The decrease in lytic activity due to Zn^2+^ was dose dependent (Figure S5). The thermal stability assay showed that when Psa was pre-incubated at 45 °C and 60 °C for 10 min, activity fell to 82% and 24% (respectively) of that seen with incubation at 37 °C (Figure 3d).

### 2.3. Bacterial Specificity of Psa

In general, phage endolysins exhibit species-specific lytic activity [9]. Therefore, we examined the species specificity of Psa against various bacteria, as shown in Table 1, using the turbidity reduction assay. We found that Psa-his was able to lyse all tested *C. perfringens* strains, although their sensitivities to Psa-his varied to some degree. The lytic activity of Psa-his was significantly increased in log growth-phase cells of *C. perfringens* SM101 compared to cells in the mid-stationary growth phase. Psa-his did not show lytic activity against the *C. perfringens* spores or any of the other tested bacteria listed in Table 1. Therefore, we infer that Psa-his is a cell-lytic enzyme with activity highly specific to *C. perfringens*.

**Figure 4:**
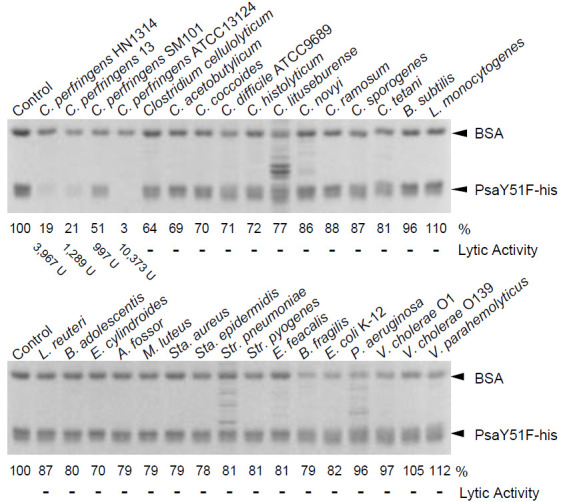
Cell binding by PsaY51F-his. Cell binding activity was measured as described in the Materials and Methods. Purified PsaY51F-his and the non-binding internal standard, bovine serum albumin (BSA) were combined with cells of a variety of bacterial species (indicated above each gel) or buffer (Control; no cells); the mixtures then were centrifuged, and the resulting supernatants were analyzed by 12.5% SDS-PAGE. Image analysis was used to quantify the intensity (in %; indicated below the gel) of the band of remaining PsaY51F-his following incubation with each bacterium; the intensity of the band in the Control (no cells) was defined as 100%. The lytic activity showed the values in Table 1.

We suspected that the species specificity of Psa-his was attributable to the C-terminal CBD. Therefore, we measured the binding activity to various bacteria using PsaY51F-his, which is a non-lytic mutant of Psa-his. As shown in Figure 4, PsaY51F-his bound only to bacteria against which Psa-his showed lytic activity. The binding of PsaY51F-his to stationary-phase *C. perfringens* SM101 was quite low, but the band intensity (indicative of the avidity of binding) was apparently lower that for bacteria against which Psa-his did not show lytic activity (Figure 4 *C. perfringens* SM101). The binding of Psa was low in stationary-phase *C. perfringens* SM101 (conditions under which Psa-his showed weak lytic activity) but was strong against logarithmic growth-phase *C. perfringens* SM101 (conditions under which Psa-his showed strong lytic activity) (Figure S6). In addition, many bands other than bovine serum albumin (BSA) and PsaY51F-his were observed in some lanes, as shown in Figure 4; these extra bands presumably reflected autolysis of these bacteria. These results indicated that the species-specific lytic activity of Psa-his depends largely on the enzyme’s binding to bacterial cells, such that the strength of Psa’s lytic activity depends on avidity of binding.

### 2.4. Synergistic Effect of Psa and the C. perfringens-Specific Endolysin Psm

**Figure 5:**
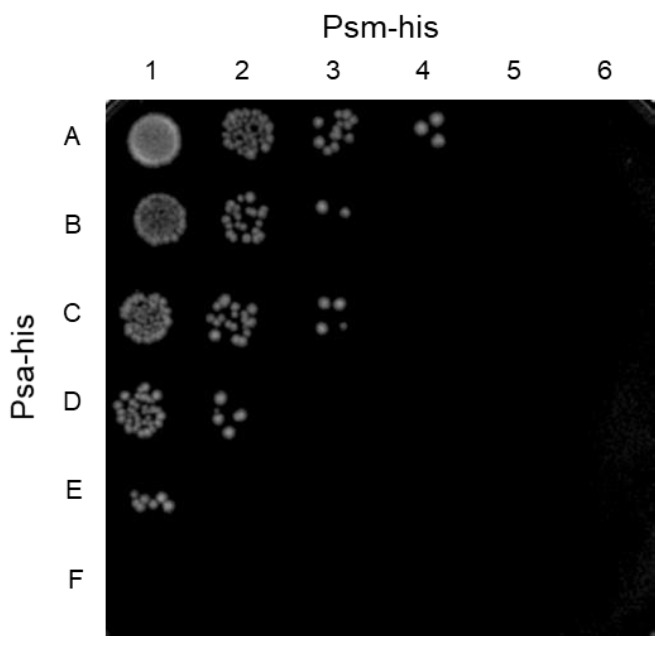
Synergistic killing by Psa-his and Psm-his of C. perfringens HN1314 in a checkerboard assay. Serial 2-fold dilutions with PBS (-) of Psa-his and Psm-his (20 μL; individually and in combination) were mixed with 20 μL (4 × 10^6^ cells) of *C. perfringens* HN1314 on ice. After a 30-min incubation at 37 °C, 2 μL (2 × 10^5^ cells) of each mixture were spotted to GAM/2 agar plates and incubated anaerobically at 37 °C for 12 h. Rows A-F are Psa-his concentrations of 4.3, 8.7, 17.3, 34.6, 69.2, and 138.5 nM. Columns 1-6 are Psm-his concentrations of 1.0, 1.9, 3.9, 7.8, 15.5, and 31 nM.

The combination of two endolysins cleaving different sites of peptidoglycan in *S. pneumoniae* has been reported to have a synergistic killing effect on the host bacterium [33]. Previously, we reported on Psm, a deduced muramidase endolysin that specifically lyses *C. perfringens* [31]. Since Psa and Psm are *C. perfringens*-specific lytic enzymes with distinct peptidoglycan cleavage sites, the simultaneous use of Psa and Psm may have a synergistic killing effect specific to *C. perfringens*. Therefore, the minimum bactericidal concentration (MBC) was measured using *C. perfringens* HN1314 with samples containing Psa and Psm, alone and together. The MBCs for Psa and Psm alone were 138.5 nM and 15.5 nM, respectively (Figure 5, spot A5 and F1). The mixtures of Psa and Psm, serially diluted at concentrations under each MBC, were tested with *C. perfringens* HN1314 to calculate the fractional inhibitory concentration (FIC) index. The most effective sample was a mixture of 34.6 nM (1.25 μg/mL) Psa and 3.9 nM (0.16 μg/mL) Psm (Figure 5 spot D3), which had an FIC index of 0.5 (synergy). The FIC indices of the mixture of 69.2 nM (2.5 μg/mL) Psa and 1.9 nM (0.08 μg/mL) Psm (Figure 5 spot E2) and the mixture of 8.7 nM (0.31 μg/mL) Psa and 7.8 nM (0.31 μg/mL) Psm (Figure 5 spot B4) were 0.625 and 0.563 (partial synergy), respectively. These results indicated that Psa-his and Psm-his have a synergistic killing effect on *C. perfringens*.

## 3. Discussion

Pathogenic *C. perfringens* is widely distributed in the intestines of animals, especially poultry. Infections with this bacterium not only cause severe disease, but also great financial loss. However, phage endolysin has potential as an alternative antibacterial agent to treat this pathogen. In this study, we surveyed the genome of *C. perfringens* st13 and identified a locus encoding a novel zinc-dependent amidase endolysin, Psa, that has an N-terminal amidase_2 domain (PF01510) and novel C-terminal CBD. Psa exhibited specific lytic activity against *C. perfringens* under physiological conditions, and its species specificity was highly dependent on CBD binding. We also showed that Psa has a synergistic lytic effect when combined with another *C. perfringens*-specific endolysin, Psm.

The alignment analysis and the Tyr51 mutation that abolishes lytic activity suggested that the structure of the amidase_2 domain of Psa is similar to that of T7 lysozyme, which employs a tyrosine residue in the catalytic center [36], and in contrast to the structure of AmiD, which employs a glutamic acid in the catalytic center [37]. Given that the detailed molecular mechanism of substrate recognition and the reaction mechanism of Psa remain unknown, it will be necessary to clarify these aspects of Psa by structural analysis and biochemical analysis using mutants, as has been the case for Psm [32] and the CD of *C. perfringens* Acp [39].

The lytic activity of Psa is specific to *C. perfringens* (Table 1). In particular, Psa exhibits stronger lytic activity against *C. perfringens* HN1314, which lacks 6 genes (including virulence factor-encoding loci) compared to that against its parent strain, *C. perfringens* st13. These results demonstrated the utility of strain HN1314 for detecting the activity of Psa safely and with high sensitivity. The basis for *C. perfringens* HN1314’s elevated susceptibility to Psa (compared to the parent strain) is unknown, but since the *cwlO* gene, which encodes a cell wall lytic endopeptidase, is the only the gene involved in cell wall degradation among the genes deleted from the parent strain, we speculate that this gene deletion is involved in this phenomenon. We also found that the susceptibility of *C. perfringens* SM101 to Psa, and the avidity of Psa binding to SM101, are decreased as the growth of the cells progresses (Table 1, Figure S6). This phenomenon also has been observed with the endolysins Psm [31] and Pal, a *S. pneumoniae*-specific endolysin [7]. The crosslinking rate is thought to change depending on the growth phase, such that the rate increases as the growth progresses. The binding of endolysins such as Psa to cells is thought to depend on the degree of crosslinking.

In zymographic analysis, the CD of Psa alone (PsaCD-his) showed lytic activity (Figure 2c), a phenomenon that we postulate is due to the high local concentration of the protein (within the gel). However, since PsaCD-his did not show lytic or binding activity in the liquid assay (Figure S3 and Figure 2d), this observation suggests that the CD is recruited to the substrate by the CBD. Furthermore, PsaY51F-his showed binding only to bacteria against which Psa-his showed lytic activity (Figure 4). These results indicated that the species specificity of Psa lytic activity depends primarily on the binding specificity of CBD. However, the main structure of the peptidoglycan of *C. perfringens* is shown in Figure 1, and in this structure and others, L,L-diaminopimelic acid (L,L-DAP) is crosslinked to the L,L-DAP of another peptide chain via glycine to form a minor structure [40]. In general, in DAP-type peptidoglycans, meso-DAP typically is used, while L,L-DAP is quite rare, and meso-DAP is crosslinked directly to the D-Ala of another peptide chain without interpeptide bridges. In addition, in many Gram-positive bacteria, the third amino acid in the peptide chain is L-Lys, which is crosslinked to the D-Ala in another peptide chain via various interpeptide bridges [41]. In addition, the peptidoglycan structures of *Clostridium* spp. other than *C. perfringens* and *Clostridioides difficile* have meso-DAP at position 3 of the peptide chain, which is directly crosslinked to D-Ala; that is, the interpeptide bridge does not contain Gly [42]. Acd24020, the endopeptidase specific to *C. difficile*, binds to many *Clostridium* spp., but does not bind to *C. perfringens* [43]. These data suggest that the peptidoglycan structure of *C. perfringens* is very distinctive and unlike that of other bacteria, even those in the same genus. We propose that the unique peptide chain of *C. perfringens* is the binding substrate for Psa and Psm, but the details of this binding mechanism are unclear and require further analysis.

Psa exhibits strong lytic activity against *C. perfringens* at a pH range of 6 to 8, at NaCl concentrations above 150 mM, and at 37 °C. These properties mean that Psa acts under physiological conditions. In addition, Psa is considered to be a zinc-dependent amidase, given that this enzyme is highly homologous to T7 lysozyme, a zinc-dependent amidase, and given that the amino acids involved in zinc binding are conserved (Figure S1). The lytic activity of Psa was not affected by EDTA, suggesting that zinc ions are strongly bound to Psa. The addition of zinc ions actually decreased Psa’s lytic activity in a concentration-dependent manner (Figure S5). Similar inhibition/reduction of the activity by excess zinc also has been reported in T7 lysozyme [36], LysH5 [44], and PlyTW [45], all of which are endolysins with a zinc-dependent amidase_2 domain. This decrease in activity by added zinc presumably reflects non-specific binding of excess zinc ions by the protein, which is a general effect of zinc ions on proteins [46]. The free zinc concentration in the living body is thought to be lower than the concentration at which the lytic activity of Psa is eliminated. Therefore, Psa is expected to be of use in living organisms as an alternative antibacterial agent.

Psm, a *C. perfringens*-specific endolysin, was expected to enhance the bactericidal activity of Psa, given that the peptidoglycan cleavage site of Psm is between *N*-acetylmuramic acid and *N*-acetylglucosamine, a site distinct from that of Psa. The MBC concentration of Psm alone (15.5 nM) was lower than that of Psa alone (138.5 nM) (Figure 5), likely because hydrolysis of the glycan chain has higher bactericidal effects than hydrolysis of the peptide chain. The strongest bactericidal effects were observed when the combination of 34.6 nM Psa and 3.9 nM Psm (Figure 5 spot D3) was employed against *C. perfringens*, providing a FIC index of 0.5. In addition, at other mixing ratios (Figure 5 spots E2 and B4), the FIC index was between 0.5 and 0.75. Therefore, Psa and Psm are considered to have a synergistic effect. These results indicate that lytic enzymes with different cleavage sites provide a higher bactericidal effect than obtained when either enzyme is used alone. If the efficacy of this endolysin combination against *C. perfringens* infection can be confirmed in vivo, these enzyme mixtures could be used in several ways. For example, spraying areas affected with gas gangrene caused by *C. perfringens* with these enzyme mixtures may be effective for treating drug-resistant *C. perfringens*. In addition, pre-mixing these enzyme mixtures into poultry feed could decrease foodborne *C. perfringens* infection. Furthermore, if this endolysin combination can be prepared in enteric capsules, the enzyme mixtures could be efficiently delivered to the intestine without being degraded by gastric acid, which would ensure the efficient treatment of *C. perfringens* intestinal infections without disturbing the normal intestinal microflora and overcome the deterioration of the intestinal environment caused by the abnormal growth of *C. perfringens*, thereby contributing to the maintenance of host health.

## 4. Materials and Methods

### 4.1. Construction of the Expression Vector

Standard PCR methods were used for the construction of the vector for expression of N-terminal histidine-tagged wild-type Psa (Psa-his). Briefly, PCR was carried out using the CPE1138-F (GGGCAT**ATG**GATATTAAGAAAGTATATTTAAAAGG) and CPE1138-R (CCCCTCGAG**TTA**AAAAATACTAAATTCACCATTA) primer pair with the genomic DNA of *C. perfringens* st13 as the template. In the primer sequences, restriction enzyme sites are underlined, and stop and start codons are indicated by bold letters. The resulting PCR products were digested with NdeI and XhoI and then cloned into a similarly digested pColdII expression vector (Takara Bio Inc., Shiga, Japan); the resulting plasmid was designated pColdII-CPE1138. To construct the vectors for overexpression of the N-terminal and C-terminal domain mutants, PsaCD-his and PsaBD-his (respectively), the same method was used but using pColdII-CPE1138 as the template with the pColdF2 (GTAAGGCAAGTCCCTTCAAGAG) and CPE1138-CD-R164 primer (GCTTTCTCGAG**TTA**ATTGTTATTAGTAACTTGAG) primer pair (for the N-terminal domain construct) and the CPE1138-CBD-F165 (CTAATCAT**ATG**ATAGATATTAAAGCTAATGC) and pColdR (TGGCAGGGATCTTAGATTCTG) primer pair (for the C-terminal domain construct). The resultant plasmids were designated pColdII-CPE1138-CD164 and pColdII-CPE1138-CWB165, respectively. Overlap extension PCR [47] was used to construct the Y51F mutant of Psa-his using the combination of primers pColdF2, pColdR, Y51F-FW (GATTTTATATGATAGGA**ttt**AATTTCTATGTACG), and Y51F-RV primer (CGTACATAGAAATT**aaa**TCCTATCATATAAAATC), with pColdII-CPE1138 as the template. For these primers, mutated bases are indicated by bold lowercase letters. The amplified DNA fragment was cloned into the pColdII vector as described above. The sequences of the PCR-amplified fragments in all constructs were verified with an ABI PRISM^®^ 3130xl using the methods recommended by the manufacturer.

### 4.2. Expression and Purification of the Proteins

*E. coli* BL21-CodonPlus-RIL strains carrying the plasmids were cultured at 37 °C in M9 medium containing 0.2% (*w/v*) glucose, 0.2% tryptone, 0.001% thiamine, and the appropriate antibiotics until the mid-log growth phase; the cultures then were incubated in ice water for 30 min. After the addition of isopropyl-β-D-thiogalactopyranoside (final concentration 1 mM), cells were further cultured at 15 °C for 20 to 24 h. Purification was carried out by the previously described method [31,39]. Briefly, the harvested cells were suspended in lysis buffer (50 mM sodium phosphate, pH 8.0, 500 mM NaCl, 10 mM imidazole, and 5 mM β-mercaptoethanol); the suspended cells were disrupted by sonication; and the lysate was subjected to immobilized metal chelate affinity chromatography using a 0.5- to 1-mL bed volume of TALON^®^ Metal Affinity Resin (Clontech Laboratories, Inc., CA, USA). Protein was eluted by two passages of 1.5–3 mL of elution buffer [50 mM sodium phosphate, pH 8.0, 250 mM imidazole, pH 8.0, 20% (*w/v*) glycerol, 1 mM β-mercaptoethanol]. The eluent from the resin was dialyzed against dialysis buffer (25 mM Tris-HCl, pH 8.0, 250 mM NaCl, 20% glycerol, 1 mM dithiothreitol) and then stored at −80 °C.

### 4.3. Turbidity Reduction Assay

Measurement of the endolysin activity was performed by the turbidity reduction assay as described previously [8,31]. The bacteria and culture conditions used in the turbidity reduction assay are noted in Table S1. Cultured cells at mid-stationary phase were harvested by centrifugation at 10,000× *g* for 5 min at 4 °C. The harvested cells were washed three times with PBS (-), and then the final OD_600_ was adjusted to 1 with the same buffer. The lytic activity was initiated by adding 100 μL of a serially diluted solution of Psa stock solution (1 mg/mL) with PBS (-) to 100 μL of cell suspension in a standard 96-well microplate on ice. The OD_600_ was monitored at 37 °C at 1-min intervals with 10-sec mixing for 35 min using a microplate reader (SpectraMax^®^ M5e Multi-Mode Microplate Readers, Molecular Devices Corp., Sunnyvale, CA, USA). After the first 5 min of incubation (pre-incubation), the constant decrease (*R*^2^ > 0.95) in OD_600_ was expressed as *V*_max_. Results of the negative control were subtracted from sample values. One unit was defined as the reciprocal of the dilution that decreased OD_600_ by 50% in 30 min; values were expressed in units of U/mL (mg).

The optimal pH, NaCl concentration, and heat stability of Psa-his were examined against *C. perfringens* HN1314 cells. For determination of the optimal pH of the activity, the assay was performed using a universal buffer (40 mM phosphoric acid, 40 mM boric acid, 250 mM NaCl, with the pH adjusted from 4 to 9 with NaOH). To investigate the effects of NaCl and metal ions on the activity, the following were used as assay buffers: (25 mM Tris-HCl, pH 7.0, 0–1000 mM NaCl, 1 mM EDTA, 1 mM dithiothreitol) and (25 mM Tris-HCl, pH 7.0, 250 mM NaCl, 1 mM metal chloride or EDTA, 1 mM dithiothreitol), respectively. The heat stability of Psa-his was tested in assay buffer (25 mM Tris-HCl, pH 7.0, 250 mM NaCl, 1 mM EDTA, 1 mM dithiothreitol) after Psa-his was pre-incubated for 10 min at 37, 45, 60, 75, and 100 ^o^C, respectively. The remaining and relative activities were calculated based on the *V*_max_. All experiments were performed in triplicate, and the results are expressed as the mean values.

### 4.4. Renaturing SDS-PAGE Analysis (Zymography)

Lytic activities of wild-type and mutant Psa-his were measured by renaturing SDS-PAGE analysis (zymography) based on autoclaved cells, as described previously [31], using *C. perfringens* HN1314 cells [38]. Bacterial cells were cultured for 8 h at 37 °C in TY-G1 medium (3% tryptone, 2% yeast extract, 1% glucose, 0.1% sodium thioglycolate) under anaerobic conditions in tightly capped tubes. The gel was stained with 1% methylene blue in 0.01% KOH and then destained, to render the clear bands (the result of lysis) more visible [48].

### 4.5. Cell Binding Assay

The cell binding assay was carried out by the following method. Bacterial strains cultured in the same manner as in the turbidity reduction assay were centrifuged and washed 3 times with PBS (-). The washed cells were suspended in the same buffer to adjust the OD_600_ to 50. An aliquot of 3 μL of the purified PsaY51F-his (1 mg/mL) was combined with 2 μL of BSA (2 mg/mL) and 5 μL of PBS (-), and the mixture was incubated with 20 μL of the 50-OD_600_ cell suspension or PBS (-) for 15 min on ice. The samples then were centrifuged at 20,000× *g* at 4 °C for 3 min. The supernatants (18 μL) were recovered and 4× SDS-PAGE sample buffer (6 μL) was added. The mixtures were heated at 95 °C for 5 min, and then 10-μL samples were applied to SDS-PAGE gels. Following staining with CBB-R250, the gels were scanned with a ChemiDoc XRS Plus System (Bio-Rad Laboratories Inc., Hercules, CA, USA) and analyzed using NIH ImageJ software (Version 1.48) (http://rsbweb.nih.gov/ij/) (2 February 2018).

### 4.6. Measurement of Synergistic Effect

To determine the minimum bactericidal concentration (MBC), the following method was used with *C. perfringens* HN1314. The cells were washed three times with PBS (-) and the OD_600_ was adjusted to 1 (approximately 2 × 10^8^ CFU/mL). Aliquots of 20 μL of cell suspension and 20 μL of serially diluted endolysins (Psa and/or Psm) were mixed and incubated at 37 °C for 30 min. Samples (2 µL) were spotted onto GAM/2 agar plates (generated by pouring agar plates using molten agar that had been diluted two-fold with GAM medium) and then incubated at 37 °C overnight under anaerobic conditions. The combined effect of Psa and Psm was measured by the checkerboard method as described below. For all of the spots on the agar plates that corresponded to an MBC, the sum of the FICs (ΣFIC) was calculated for each spot using the equation ΣFIC = FIC_Psa-his_ + FIC_Psm-his_ = (C_Psa-his_/MBC_Psa-his_) + (C_Psm-his_/MBC_Psm-his_), where MBC_Psa-his_ and MBC_Psm-his_ are the MBCs of Psa-his and Psm-his alone, respectively, and C_Psa-his_ and C_Psm-his_ are the concentrations of the endolysin in combination, respectively [49]. Results for synergy testing were interpreted as follows: FIC of <0.50, synergy; FIC 0.51 to 0.75, partial synergy; FIC 0.76 to 4.00, indifference; and FIC >4.00, antagonism [49].

## Figures and Tables

**Table 1:** Lytic spectrum of Psa-his.

Organism	Strain	Relative Activity (%)
Gram(+)		
*Clostridium perfringens*	HN1314	100 (3967 U)
*Clostridium perfringens*	st13	32 (1289 U)
*Clostridium perfringens*	ATCC13124	261 (10,373 U)
*Clostridium perfringens*	SM101	25 (997 U)
*Clostridium perfringens*	SM101 (4 h)	325 (12,911 U)
*Clostridium perfringens*	SM101 (spore)	-
*Clostridium acetobutylicum*	ATCC824	-
*Clostridium cellulolyticum*	ATCC35319	-
*Clostridium coccoides*	ATCC29236	-
*Clostridium histolyticum*	ATCC19401	-
*Clostridium lituseburense*	ATCC25759	-
*Clostridium novyi*	ATCC17861	-
*Clostridium ramosum*	ATCC25582	-
*Clostridium sporogenes*	ATCC3584	-
*Clostridium tetani*	KZ1113	-
*Clostridioides* *difficile*	ATCC9689	-
*Atopobium fossor*	ATCC43386	-
*Bacillus subtilis*	168	-
*Bifidobacterium adolescentis*	ATCC15703	-
*Enterococcus faecalis*	IID682	-
*Eubacterium cylindroides*	ATCC27805	-
*Lactobacillus reuteri*	ATCC23272	-
*Listeria monocytogenes*	HCIPH A5-1	-
*Micrococcus luteus*	ATCC4698	-
*Staphylococcus aureus*	ATCC6538P	-
*Staphylococcus epidermidis*	ATCC35984	-
*Streptococcus pneumoniae*	IID555	-
*Streptococcus pyogenes*	124/0207	-
Gram(−)		
*Bacteroides fragilis*	ATCC25285	-
*Escherichia coli*	K-12	-
*Pseudomonas aeruginosa*	PAO1	-
*Vibrio cholerae*	O1/P1418	-
*Vibrio cholerae*	O139/MDO-6	-
*Vibrio parahaemolyticus*	RIMD2210115	-

The lytic activities of enzymes were determined by the turbidity reduction assay, using mid-stationary growth-phase cells of various bacteria, and log growth-phase (4 h) cells and spores (spore) of *C. perfringens* SM101. Spores were prepared by culturing in mDS for 48 h followed by sonication, washing three times with phosphate-buffered saline without Ca and Mg (PBS(-)), and counting using a Thoma cell counter. The culture conditions and media are indicated in Supplementary File Table S1. The cell suspension in PBS(-) (1 OD_600_) was mixed with Psa (10 μg/mL). The relative activities were obtained by normalizing to that against *C. perfringens* HN1314 cells, which was defined as 100%. -: No lytic activity was detected.

## Data Availability

The data presented in this study are available on request from the corresponding author.

## Supplementary Materials

The following are available online at https://www.mdpi.com/2079-6382/10/3/245/s1, Figure S1 Alignment of the catalytic domain T7 (PF01510: Amidase_2) family amidase. Figure S2 Genes flanking *psa* (CPE1138) in the *C. perfringens* st13 genome. Figure S3 Lytic activities of Psa-his, PsaCD-his, PsaBD-his, and PsaY51F-his. Figure S4 Zymography analysis of Psa-his, PsaCD-his, PsaBD-his, and PsaY51F-his. Figure S5 Inhibition of Psa-his by Zn^2+^. Figure S6 Binding of Psa-his to *C. perfringens* SM101 cells and spores. Table S1 Strains and culture conditions used in this study.

## Abbreviations

BLAST	basic local alignment search tool
BSA	bovine serum albumin
CBD	cell wall binding domain
CD	catalytic domain
DAP	diaminopimelic acid
EDTA	ethylenediaminetetraacetic acid
FIC	fractional inhibitory concentration
OD_600_	optical density at a wavelength of 600 nm
PBS (-)	phosphate-buffered saline without Ca and Mg
SDS	sodium dodecyl sulfate
SDS-PAGE	SDS polyacrylamide gel electrophoresis
