# Supplementary material for: A Putative Amidase Endolysin Encoded by Clostridium perfringens St13 Exhibits Specific Lytic Activity and Synergizes with the Muramidase Endolysin Psm
Source: Antibiotics (Basel). 2021 Mar 1;10(3):245. doi: 10.3390/antibiotics10030245 (PMC7999503; doi:10.3390/antibiotics10030245)
Supplement: Supplementary file 1 [file antibiotics-10-00245-s001.pdf]

Supplementary Materials:

A putative amidase endolysin encoded by *Clostridium perfringens* st13 exhibits specific lytic activity and synergizes with the muramidase endolysin Psm

Hiroshi Sekiya, Maho Okada, Eiji Tamai, Toshi Shimamoto, Tadashi Shimamoto, Hirofumi Nariya

Figure S1 Alignment of the catalytic domain T7 (PF01510: Amidase_2) family amidase.
Figure S2 Genes flanking *psa* (CPE1138) in the *C. perfringens* st13 genome.
Figure S3 Lytic activities of Psa-his, PsaCD-his, PsaBD-his, and PsaY51F-his.
Figure S4 Zymography analysis of Psa-his, PsaCD-his, PsaBD-his, and PsaY51F-his.
Figure S5 Inhibition of Psa-his by Zn^{2+}.
Figure S6 Binding of Psa-his to *C. perfringens* SM101 cells and spores.
Table S1 Strains and culture conditions used in this study

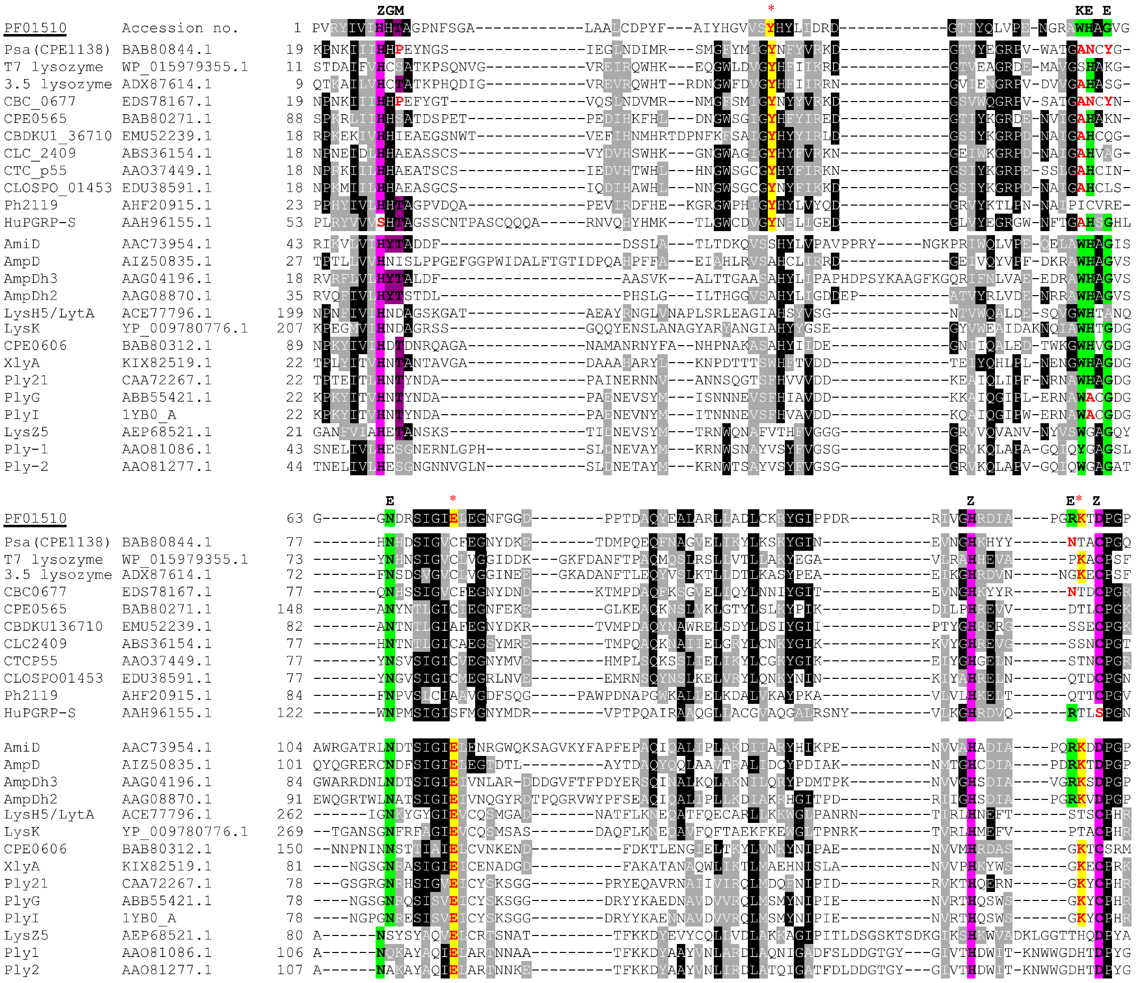

Figure S1. Alignment of the catalytic domain T7 (PF01510: Amidase_2) family amidase. Sequence alignment was created using Clustal Omega (https://www.ebi.ac.uk/Tools/msa/clustalo/). Accession no.: NCBI GenBank accession number. Conserved and identical residues (more than half) are shaded in gray and black, respectively. *: catalytic residue, Z : zinc-binding residue, G: GlcNAc-binding residue, M: MurNAc-binding residue, E: γ-D-Glu (Gln)-binding residue, and K: L-Lys-binding residue.

Amidase catalytic site

Cheng X, Zhang X, Pflugrath JW, Studier FW. (1994)The structure of bacteriophage T7 lysozyme, a zinc amidase and an inhibitor of T7 RNA polymerase. Proc Natl Acad Sci U S A. 91(9):4034-4038.

Zn binding residues

Low LY, Yang C, Perego M, Osterman A, Liddington RC. (2005) Structure and lytic activity of a Bacillus anthracis prophage endolysin. J Biol Chem. 280(42):35433-35439.

Substrate binding site

Guan R, Roychowdhury A, Ember B, Kumar S, Boons GJ, Mariuzza RA. (2004) Structural basis for peptidoglycan binding by peptidoglycan recognition proteins. Proc Natl Acad Sci U S A. 101(49):17168-17173

a

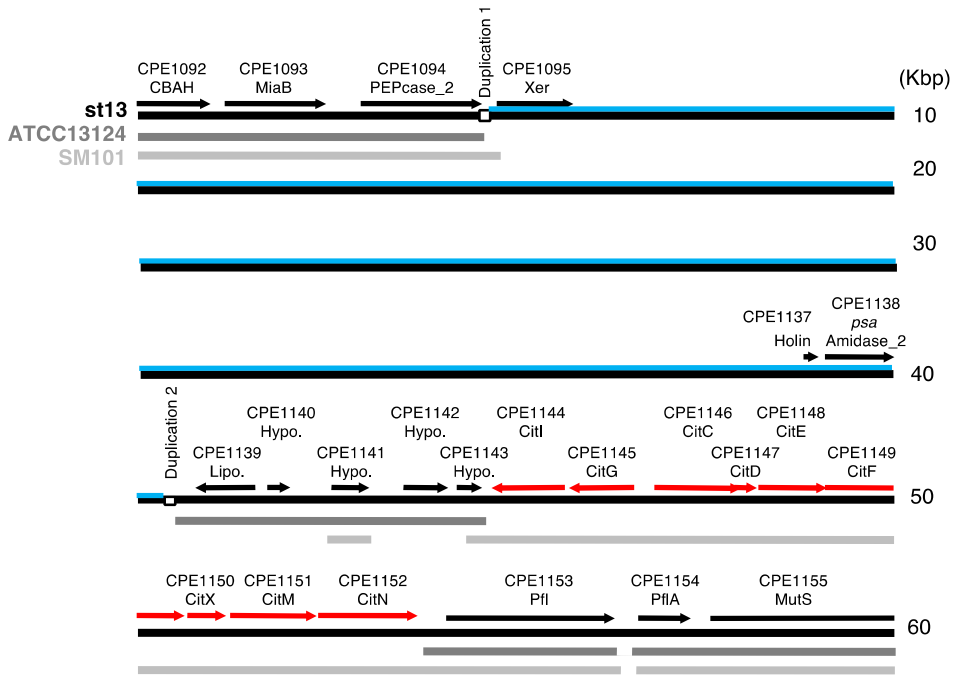

b

Gene Tag	aa	Description
CPE1095	338	Phage_integrase/Site-specific recombinase, Xer
CPE1096	99	Hypo.
CPE1097	70	HTH_XRE
CPE1098	154	Hypo.
CPE1099	100	Hypo.
CPE1100	230	HTH_XRE
CPE1101	77	Hypo.
CPE1102	276	Hypo.
CPE1103	1064	Cons., Phage-related
CPE1104	143	Cons.
CPE1105	106	Hypo.
CPE1106	56	Hypo.
CPE1107	306	Cons.
CPE1108	60	Hypo.
CPE1109	151	Hypo.
CPE1110	365	Bro-N, Prophage antirepressor
CPE1111	52	Hypo.
CPE1112	88	Toxin YafO, type II toxin-antitoxin system
CPE1113	127	Hypo.
CPE1114	59	Hypo.
CPE1115	80	Hypo.
CPE1116	136	HNH endonuclease
CPE1117	194	Phage terminase, small subunit, TerS
CPE1118	558	Phage terminase, large subunit, TerL
CPE1119	57	Hypo.
CPE1120	407	Phage_portal HK97 family
CPE1121	200	Phage prohead protease HK97 family
CPE1122	378	Phage major capsid protein, HK97 family
CPE1123	97	Head-Tail Connector Protein, HK97 family
CPE1124	105	Phage head-tail joining protein
CPE1125	147	Phage protein, HK97 family
CPE1126	116	Phage protein
CPE1127	199	Phage major tail protein, phi13 family
CPE1128	101	Phage protein
CPE1129	111	Cons.
CPE1130	933	Phage tail tape measure protein
CPE1131	231	Phage tail protein
CPE1132	983	Phage endopeptidase/lysozyme tail
CPE1133	624	Phage minor structural protein, choline-binding
CPE1134	648	Phage protein
CPE1135	80	Hypo.
CPE1136	59	Hemolysin XhlA family
CPE1137	67	Cons. Phage exported protein, Holin
CPE1138	304	Amidase_2, Endolysin

Figure S2. Genes flanking psa (CPE1138) in the *C. perfringens* st13 genome. (a) Straight lines represent the genomes of *C. perfringens* st13 (black), ATCC13124 (dark gray), and SM101 (light gray). The arrows indicate the genes (open reading frames), and the Gene Tags and names of the putative functions or protein products are indicated above the arrows. The region between duplication 1 and 2 (white square) of the *C. perfringens* st13 genome is considered a phage remnant region (light blue), given the existence of many phage-related genes. The other *C. perfringens* strains lack the phage remnant region upstream of psa. Genes related to citrate fermentation are indicated by red arrows. (b) The genes in the genome from region CPE1095 to CPE1138 in *C. perfringens* st13 are listed. Cons.: conserved protein, Hypo.: hypothetical protein. Lipo.: lipoprotein.

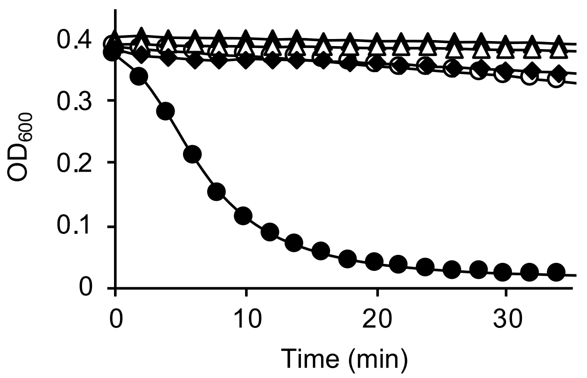

Figure S3. Lytic activities of Psa-his, PsaCD-his, PsaBD-his, and PsaY51F-his. The lytic activities of the purified enzymes were determined by the turbidity reduction assay using *C. perfringens* HN1314 cells suspended in PBS(-). Purified Psa-his (black circles), PsaCD-his (white circles), PsaBD-his (black triangles), and PsaY51F-his (white triangles) were added to pre-incubated cells to a final concentration of 10 µg/ml, and then OD_{600} was measured at 2-min intervals for 35min. A parallel no-enzyme control experiment was performed in the same manner using buffer control (black diamonds).

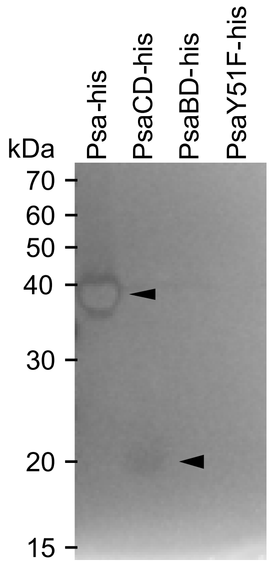

Figure S4. Zymography analysis of Psa-his, PsaCD-his, PsaBD-his, and PsaY51F-his. Purified Psa-his, PsaCD-his, PsaBD-his, and PsaY51F-his (each 2 µg) were electrophoresed in a 12.5% SDS-polyacrylamide gel containing 20 OD_{600} of heat-inactivated and SDS-treated *C. perfringens* HN1314 cells. Zymography analysis was carried out as described previously [31]. The arrowheads indicate cell lysis.

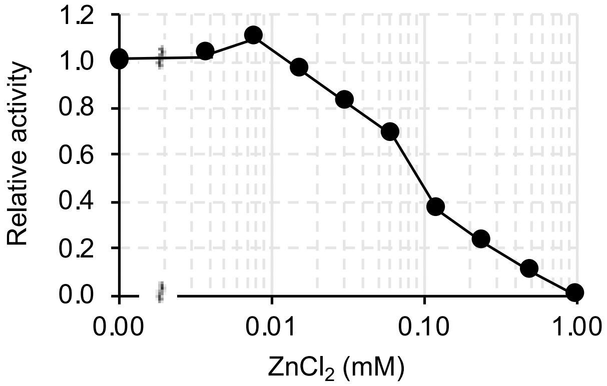

Figure S5. Inhibition of Psa-his by Zn^{2+}. Psa-his was tested at various concentrations of $ZnCl_2$ against *C. perfringens* HN1314 in 25 mM Tris-HCl pH 7.0, 250 mM NaCl, and 1 mM dithiothreitol.

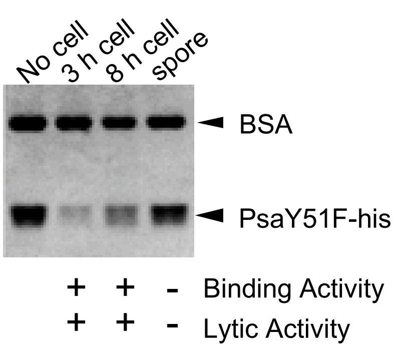

Figure S6. Binding of Psa-his to *C. perfringens* SM101 cells and its spores. The binding activity was measured according to the Materials and Methods. Purified PsaY51F-his and BSA were mixed with mid-logarithmic growth phase *C. perfringens* cells (3 h cell) or mid-stationary growth phase *C. perfringens* cells (8 h cell) or its spores (spore) or buffer (No cell), centrifuged, and the supernatants were analyzed by 12.5% SDS-PAGE. The spores of *C. perfringens* SM101 were prepared by culturing in mDS for 48 h, sonicating, washing three times with PBS(-), and counting in a Thoma cell counter.

Table S1 Strains and culture conditions used in this study.

Organism	Strain	Culture conditions
Gram(+)		
Clostridium perfringens	HN1314	AN, Static, 37°C, 8 h, TY-G1
Clostridium perfringens	st13	AN, Static, 37°C, 8 h, TY-G1
Clostridium perfringens	ATCC13124	AN, Static, 37°C, 8 h, TY-G1
Clostridium perfringens	SM101	AN, Static, 37°C, 4h and 8 h, TY-G1
Clostridium perfringens	SM101(spore)	AN, Static, 37°C, 48 h, mDS
Clostridium acetobutylicum	ATCC824	AN, Static, 37°C, 24 h, TY-G1
Clostridium cellulolyticum	ATCC35319	AN, Static, 37°C, 24 h, TY/2-CB0.5
Clostridium coccoides	ATCC29236	AN, Static, 37°C, 10 h, TY-G1
Clostridium histolyticum	ATCC19401	AN, Static, 37°C, 12 h, TY-G1
Clostridium lituseburense	ATCC25759	AN, Static, 37°C, 8 h, TY-G1
Clostridium novyi	ATCC17861	AN, Static, 37°C, 14 h, TY-G1
Clostridium ramosum	ATCC25582	AN, Static, 37°C, 12 h, TY-G1
Clostridium sporogenes	ATCC3584	AN, Static, 37°C, 8 h, TY-G1
Clostridium tetani	KZ1113	AN, Static, 37°C, 8 h, TY-G1
Clostridioides difficile	ATCC9689	AN, Static, 37°C, 8 h, TY-G1
Atopobium fossor	ATCC43386	AN, Static, 37°C, 24 h, GAM
Bacillus subtilis	168	AE, Shaking, 37°C, 8 h, LB
Bifidobacterium adolescentis	ATCC15703	AN, Static, 37°C, 10 h, TY-G1
Enterococcus faecalis	IID682	AE, Shaking, 37°C, 6 h, BHI
Eubacterium cylindroides	ATCC27805	AN, Static, 37°C, 8 h, TY-G1
Lactobacillus reuteri	ATCC23272	AN, Static, 37°C, 8 h, MRS
Listeria monocytogenes	HCIPH A5-1	AE, Shaking, 37°C, 8 h, BHI
Micrococcus luteus	ATCC4698	AE, Shaking, 37°C, 14 h, LB
Staphylococcus aureus	ATCC6538P	AE, Shaking, 37°C, 8 h, LB
Staphylococcus epidermidis	ATCC35984	AE, Shaking, 37°C, 8 h, BHI
Streptococcus pneumoniae	IID555	AE, Static, 37°C, 8 h, BHI
Streptococcus pyogenes	124/0207	AE, Static, 37°C, 8 h, BHI
Gram(-)		
Bacteroides fragilis	ATCC25285	AN, Static, 37°C, 16 h, TY-G1
Escherichia coli	K-12	AE, Shaking, 37°C, 8 h, LB
Pseudomonas aeruginosa	PAO1	AE, Shaking, 37°C, 8 h, LB
Vibrio cholerae	O1/P1418	AE, Shaking, 37°C, 8 h, LB
Vibrio cholerae	O139/MDO-6	AE, Shaking, 37°C, 8 h, LB
Vibrio parahaemolyticus	RIMD2210115	AE, Shaking, 37°C, 8 h, LB

AN and AE are aerobic and anaerobic conditions, respectively.

AN : Static culture under anaerobic conditions in tightly capped tubes

AE : Culture in test tube, shaking at 160/min

Media:

BHI: Brain Heart Infusion broth (BHI ; Eiken Chemical Co., Ltd., Tokyo, Japan)

GAM: Gifu Anaerobic Medium broth (Nissui Seiyaku Co., Ltd., Tokyo, Japan)

LB: Luria–Bertani broth (Wako Pure Chemical Industries Ltd., Osaka, Japan)

mDS (modified Duncan-Strong medium): 1.5% BACT-Peptone, 1% Na_2HPO_4 $7H_2O$, 0.4% BACT-Yeast Extract, 0.4% raffinose, 0.1% sodium thioglycolate, pH 7.8

MRS: de Man Rogosa and Sharpe broth (Sigma Aldrich, St. Louis, MO, USA)

TY-G1: 3% Tryptone, 2% Yeast Extract, 0.1% sodium thioglycolate, and 1% glucose (30)

TY/2-CB0.5: 1.5% Tryptone, 1% Yeast Extract, 0.05% sodium thioglycolate, and 0.5% cellobiose
